# Utility of Repeated Praziquantel Dosing in the Treatment of Schistosomiasis in High-Risk Communities in Africa: A Systematic Review

**DOI:** 10.1371/journal.pntd.0001321

**Published:** 2011-09-20

**Authors:** Charles H. King, Stephanie K. Olbrych, Margaret Soon, Mendel E. Singer, Jen Carter, Daniel G. Colley

**Affiliations:** 1 Center for Global Health and Diseases, Case Western Reserve University School of Medicine, Cleveland, Ohio, United States of America; 2 Department of Epidemiology and Biostatistics, Case Western Reserve University School of Medicine, Cleveland, Ohio, United States of America; 3 Schistosomiasis Consortium for Operational Research and Evaluation, University of Georgia, Athens, Georgia, United States of America; 4 Center for Tropical and Emerging Global Diseases, University of Georgia, Athens, Georgia, United States of America; 5 Department of Microbiology, University of Georgia, Athens, Georgia, United States of America; Swiss Tropical and Public Health Institute, Switzerland

## Abstract

**Background:**

Controversy persists about the optimal approach to drug-based control of schistosomiasis in high-risk communities. In a systematic review of published studies, we examined evidence for incremental benefits from repeated praziquantel dosing, given 2 to 8 weeks after an initial dose, in *Schistosoma*-endemic areas of Africa.

**Methodology/Principal Findings:**

We performed systematic searches of electronic databases PubMed and EMBASE for relevant data using search terms ‘schistosomiasis’, ‘dosing’ and ‘praziquantel’ and hand searches of personal collections and bibliographies of recovered articles. In 10 reports meeting study criteria, improvements in parasitological treatment outcomes after two doses of praziquantel were greater for *S. mansoni* infection than for *S. haematobium* infection. Observed cure rates (positive to negative conversion in egg detection assays) were, for *S. mansoni*, 69–91% cure after two doses *vs.* 42–79% after one dose and, for *S. haematobium*, 46–99% cure after two doses *vs.* 37–93% after a single dose. Treatment benefits in terms of reduction in intensity (mean egg count) were also different for the two species—for *S. mansoni*, the 2-dose regimen yielded an weighted average 89% reduction in standardized egg counts compared to a 83% reduction after one dose; for *S. haematobium*, two doses gave a 93% reduction compared to a 94% reduction with a single dose. Cost-effectiveness analysis was performed based on Markov life path modeling.

**Conclusions/Significance:**

Although schedules for repeated treatment with praziquantel require greater inputs in terms of direct costs and community participation, there are incremental benefits to this approach at an estimated cost of $153 (*S. mansoni*)–$211 (*S. haematobium*) per additional lifetime QALY gained by double treatment in school-based programs. More rapid reduction of infection-related disease may improve program adherence, and if, as an externality of the program, transmission can be reduced through more effective coverage, significant additional benefits are expected to accrue in the targeted communities.

## Author Summary

Infection by *Schistosoma* worms causes serious disease among people who live in areas of Africa, South America, and Asia where these parasites are regularly transmitted. Although yearly treatment with the drug praziquantel is fairly effective in reducing or eliminating active infection, it does not cure everyone, and reinfection remains a continuing problem in high-risk communities. Studies have suggested that a repeat dose of praziquantel, given 2 to 8 weeks after the first dose, can improve cure rates and reduce remaining intensity of infections in population-based programs. Our systematic review of published research found that, on average, in Africa, such repeated dosing appears to offer particular advantages in the treatment of *S. mansoni*, the cause of intestinal schistosomiasis, but there was less consistent improvement after double-dosing for *S. haematobium*, the cause of urogenital schistosomiasis. Based on this evidence, we used a calibrated life-path model to predict the costs and benefits of a single-dose vs. a double-dose strategy in a typical high-risk community. Our projections suggest cost-effective incremental benefits from double dosing in terms of i) limiting a person's total years spent infected and ii) limiting the number of years they spend with heavy infection, with consequent improvements in quality of life.

## Introduction

Schistosomiasis remains a significant health burden for many parts of the world, particularly where health resources are most limited [1]. Of the 239 million people with active *Schistosoma* infection in 2009 [2], 85% lived in sub-Saharan Africa, where an estimated 150,000 deaths/year were attributable to schistosomiasis [3]. Although praziquantel has been available as an effective treatment for *Schistosoma* infection for nearly 30 years [4], it is only recently that national schistosomiasis control programs have begun to distribute praziquantel widely on a population-based, mass treatment basis [5]–[7]. Of note, praziquantel treatment may not be fully curative, and questions remain about the best possible timing and frequency of praziquantel dosing for optimal control of infection and morbidity. It has been observed in some studies that repeated praziquantel dosing can improve the treatment-associated reductions in worm burden and also increase its overall effectiveness for parasitological cure. Program policy planners have asked whether such double dosing would offer advantages in aggressive population-based programs aiming to fully minimize levels of infection and infection-associated morbidity, especially for high risk locations where transmission is not effectively interrupted by mass drug delivery [8], [9]. It is hypothesized that, in such communities, repeat dosing at a 2–8 week interval might be more effective, in part by addressing the relative resistance of immature schistosomes to praziquantel at 14–35 days after infection [10]. This would be relevant if transmission is ongoing or has occurred recently, and repeated dosing might then serve to reduce infection prevalence and intensity more effectively among frequently exposed persons [11] by treating the initially immature forms after they had matured (during the treatment interval) into drug-susceptible adult worms [10].

The study reported here is a systematic review of population-based studies that compare single- *vs.* repeated-dose praziquantel treatment of *Schistosoma mansoni* or *S. haematobium* in high-risk locations in Africa [12]–[21]. Comparisons of treatment efficacy in terms of cure and reduction of infection intensity are reported. Because reinfection remains an ongoing challenge for schistosomiasis control programs [22], the projected costs and long-term impacts of implementing either of these two strategies are provided in a cost-effectiveness analysis that models the probable lifetime experience of treated and untreated residents in a ‘problem’ community setting where schistosomiasis is highly endemic (i.e., >50% infection prevalence among school age children [23]), and risk of reinfection remains high despite treatment intervention [9].

## Methods

### Systematic review for data on repeated treatment outcomes

Following a pre-established protocol, we performed systematic searches of electronic databases PubMed, UnboundMedline, and EMBASE for relevant studies using the search terms ‘schistosomiasis’, ‘dosing’ and ‘praziquantel’. Hand searches were also performed of personal collections and of the bibliographies of recovered articles, limiting our search to articles in English published after the year 1982. Candidate studies obtained by these searches were abstracted into a study database, and each was reviewed for relevance by three experienced readers [24].

### Inclusion/exclusion criteria

Studies included in this systematic review had to involve results for both single and double praziquantel treatment for either *Schistosoma mansoni* or *Schistosoma haematobium* infection, involve population-based or sub-population (*e.g.*, schools)-based drug treatment, and provide technical details on i) the diagnostic techniques used to define infection status, ii) the drug dosing tested, and iii) the interval for follow up. Study reports also had to provide location, study size, targeted age groups and sufficient treatment outcomes data to allow calculation of per treatment cure rates and rates for reduction of infection intensity (as measured by proportional reductions in egg output in standardized testing). Studies that involved non-praziquantel drugs (only) or that lacked these essential components were excluded, as were studies in which the interval between repeated doses was <2 weeks or >8 weeks. For each included study, additional information was extracted on study design (RCT or observational), location, local pre-control prevalence of schistosomiasis, and study size.

### Data analysis

There was considerable variation detected in study outcomes among the combined data sets for the two *Schistosoma* parasite species. The proportion of total variation in pooled study estimates was quite high, most likely due to genuine differences between locations. As measured by Higgins's and Thompson's *I^2^* statistic for heterogeneity [25], [26], 79% of differences in odds of cure after one *vs.* two doses was due to between-study heterogeneity, and not just chance. Significant heterogeneity was also observed among studies of *S. haematobium* having different levels of pre-treatment prevalence of infection (for cure ORs, *I^2^* = 85%) Because of this, we elected not to use meta-analytic techniques to calculate pooled estimates for overall cure rates or the relative impact of treatment on infection intensity. Instead, in this report we elected to summarize the range of observed outcomes, stratified according to species and pre-treatment prevalence, and then explore the impact of these differences in projected long-term program outcomes using life-path modeling for residents of high-risk communities.

### Markov decision-tree modeling and cost-effectiveness analysis

In order to extend the review's findings in terms of evidence-based policy prediction, life-path Markov simulations (including sensitivity analysis based on the range of observed outcomes) was used to estimate the relative benefits and incremental cost-effectiveness of long-term double *vs.* single praziquantel treatment strategies in a typical *Schistosoma*-endemic setting. The decision-analysis Markov model followed the yearly experience of a cohort of persons from the age of 5 yr until death or until life expectancy (age 60 yr). Transitions among infection states (uninfected, light, or heavy infection) from year to year were based on conditional probabilities derived from field data (see Tables S1 and S2). The model then summed, by year, the cumulative life years, quality-adjusted life years, infected years, infectious burden, and the cost of therapy given, if any, for the modeled cohort depending on whether single-dose therapy, double-dose therapy or no therapy was given. These modeling simulations were conducted using Tree-Age Pro 2009 Software (version 1.0.2, TreeAge Software, Inc. Williamstown MA). [The interested reader can review and modify an example model from the study, which is provided in full in Model S1. This contains a compressed file of the Markov model (and its necessary input tables) developed for modeling community-based treatment of *S. haematobium*. Upon request, the corresponding author (CHK) can also provide the related model for school age treatment of *S. haematobium*, and the two corresponding models developed for *S. mansoni* control.]

As constructed, the model used age-specific data on infection and reinfection to predict the *lifetime* impact of three basic treatment strategies, (i) no treatment (i.e., the default baseline for comparison); (ii) single annual dosing of PZQ; or (iii) annual delivery of two doses of PZQ separated by 2–8 weeks. These were also implemented either as a school-based program (school age children (5–15 yr) only, the age group most susceptible to heavy infection [15], [27]) or a community-based program (treating children and adults), in order to contrast the relative impact of these two approaches. The effect of incomplete coverage was estimated by identifying and subsequently tracking subgroups who missed treatment in any given year.

Consistent with current deworming program practice, neither of our modeled PZQ strategies used individual level diagnostic testing to assign subject treatment. Rather, each simulation followed a comprehensive (or school age-targeted) blanket treatment program. To simulate the long-term impact of each strategy, the model was designed to follow a cohort of people at risk from age 5 to age 60 by simulating 55 successive one-year exposure/treatment cycles in an endemic area. Based on participation levels, treatment received, and risk for reinfection, the termination of each yearly cycle allowed individuals to transition to a heavy, light, or no infection status in the next year, or to die from schistosomiasis or from another competing cause. Because disease burden is proportionate to intensity of infection, the model tracked and distinguished those with heavy infection from those with light infection and assigned a different annual disease burden score to each of these infection states. In addition, the model tracked average ‘cumulative egg years’ (cumulative egg output/year over 55 years) as individuals transitioned through heavy infection, light infection, and uninfected states during the course of their 55 annual cycles. Other relevant model assumptions are inventoried in Table 1.There was no gender restriction on the model population, as both genders were considered susceptible to the infection and its morbidity.

**Table 1:** Modeling assumptions for cost-effectiveness analysis.

1) All 5-year-old children will be eligible for treatment.
2) There are no persistent adverse side effects of the medication.
3) The willingness to pay is set at one gross domestic product (GDP) per capita of Kenya as of 2010, $1600.
4) There is no major variation of schistosomiasis transmission in the target population over time.
5) Transmission rates are unaffected if there still exist untreated children who can perpetuate contamination.
6) Risks of competing mortality, not related to schistosomiasis, are even across each treatment strategy and infection level.
7) Children have not previously received treatment, and do not receive treatment outside the program.
8) The detriment associated with infection and specifically with heavy infection is the same for all ages.

### Markov inputs

For the Markov model, relevant ranges of treatment participation and outcomes parameters used in the model (Table S1) were obtained from our systematic review. Risk of reinfection following treatment (Tables S1 and S2) was based on multi-year drug treatment experience in a highly endemic area of Kenya [28]–[30]. Sensitivity analysis for model outcomes was completed for all discrete variables but necessarily excluded table-based variables. The ranges used for the sensitivity analysis of these parameters are, where possible, the 95% confidence interval of the original data source. Where it was not possible to obtain a 95% confidence interval, the parameter values were allowed to range from 0.5 to 2 times the base estimate. References listed in Table S1 [1]–[3], [5], [12], [16], [17], [21], [29], [31], [33]–[35], [53]–[59] indicate the different sources of the base estimates and the range of each input value for the model.

The cost of treatment was determined by inputting estimated the cost of each pill multiplied by the number of pills needed at each age (based on average weight) and the cost of delivery of the drug. The cost per pill for bulk purchased generic praziquantel was obtained from recent literature [5]. The cost of delivery of treatment is estimated using the median financial cost of similar public health projects for mass treatment of lymphatic filariasis [31] and for community-directed treatment of onchocerciasis in Cameroon, Nigeria, and Uganda (McFarland, D, *personal communication*). The base estimate of the cost of drug delivery was $0.811 (Table S1) which reflected the financial cost per treatment without including donated volunteer time. Full economic costs, including donated time and resources, were estimated to be as high as $5.82 for some areas [31].

### Calculating incremental cost-effectiveness of a second dose of praziquantel

For this phase of the analysis, cumulative time-discounted costs were summed for an average individual over the 55 year period simulated in the model, based on his or her year-to-year participation. For example, areas with lower participation had lower average cumulative costs, and school-age programs had lower costs than community wide programs that offered adult coverage. Impact of therapy was measured in terms of reduction in the number of years spent infected, and reduction in cumulative intensity of infection during the 5–60 year old period of life (cumulative egg-years). We also estimated the benefit of therapy on quality of life by summing cumulative Quality-Adjusted Life-Years, or QALYs [32], based on time spent with heavy infection, light infection or uninfected. Here, base estimates of utility of moderate-heavy infection (0.9 QALY) and of light infection (0.986 QALY) were adapted from recent evidence-based estimates of schistosomiasis-related disability [33]–[35]. The comparisons of primary interest were the incremental cost-effectiveness ratios (ICERs) for those who were offered 0, 1, or 2 doses of PZQ per year. These represented, respectively, areas with no control, areas with annual treatment with a single PZQ dose, and areas with annual treatment with repeat PZQ treatment 2–8 weeks after the initial round of treatment. In keeping with standard practice for health-related cost-effectiveness analysis, we performed analysis from the societal perspective and all future costs and utilities (QALYs) were time discounted at 3% per annum [32].

## Results

### Studies identified and their characteristics

**Figure 1:**
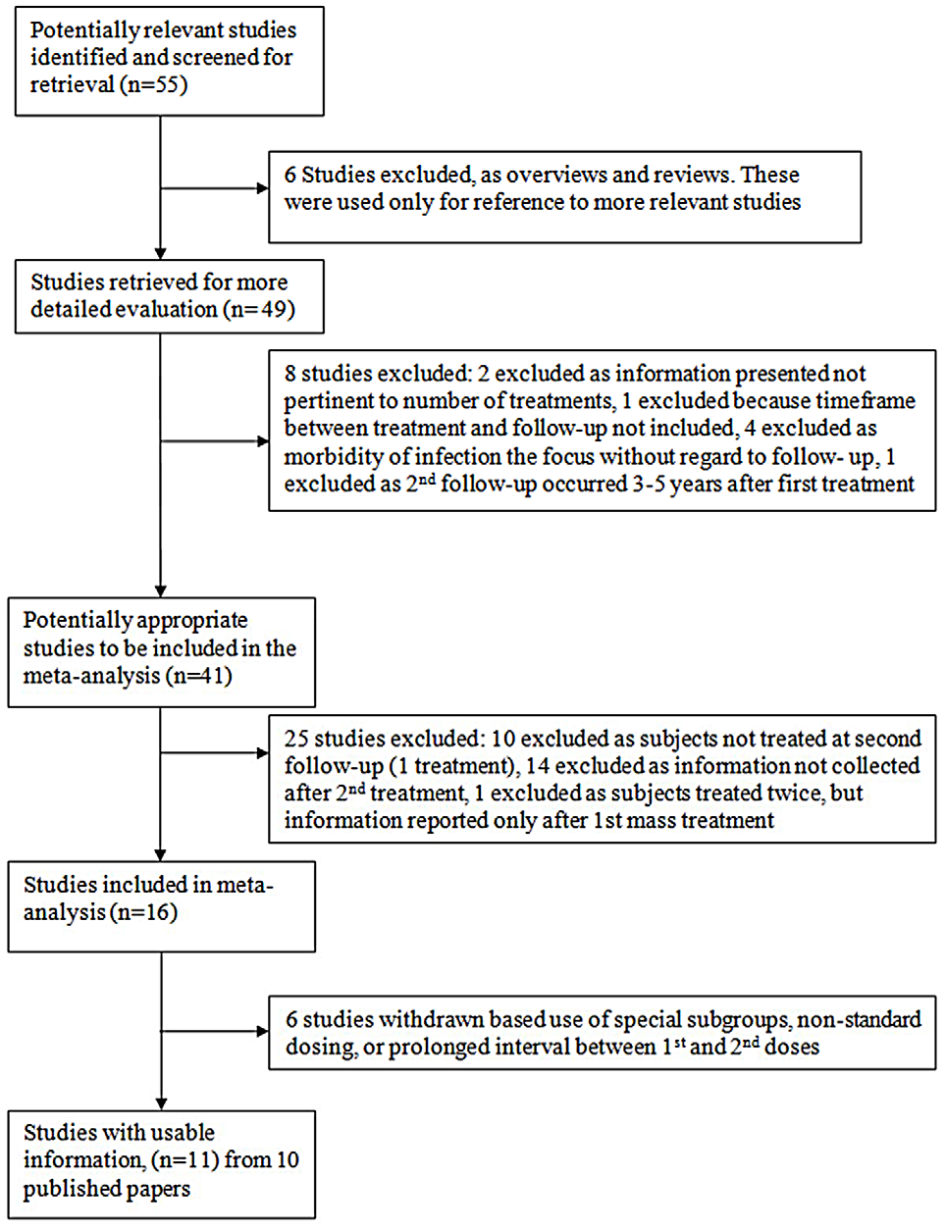
Flow diagram of the study selection process for inclusion in this paper's systematic review. Overview of the review process for papers reporting on the efficacy of praziquantel repeat dosing for treatment of *S. haematobium* or *S. mansoni* infection in Africa. Shown are the reasons for exclusion/inclusion at each step of the systemic review.

Initially, fifty-five studies were identified by database searches (complemented by hand searches) for potential inclusion in this systematic review (Figure 1). Of these, 41 reported on population-based or school age schistosomiasis treatment interventions that were the focus of our analysis. However, 25 were eventually excluded due to lack of information on the final outcomes of the double treatment group, or because the doses or dosing intervals used were not relevant to our study. A total of 10 papers, reporting studies from 11 locations across Africa, were included for analysis (Tables 2 and 3). These studies were published between 1998 and 2010, suggesting relevance to current treatment initiatives. There were 5 studies reporting on cure rates (Table 2) as well as reduction in egg output (Table 3) for *S. mansoni* infections, and 6 studies reporting on these outcomes after treatment for *S. haematobium*. Also, for the two parasite species, three studies (each) described the relative treatment impact for persons having heavy *vs.* light infection intensity (Tables 2 and 3). Although local infection prevalence was not a criterion for selection, all of the populations for the included studies had either moderate (>40%) or high prevalence (>50%) [23] of *Schistosoma* infection before therapy was given.

**Table 2:** Observed cure rates for schistosomiasis in selected field studies.

Study [citation]	Country Location	Study Size	Diagnosed with	Local Pre-treatment Prevalence	Two Dose Interval	Follow up interval	One Dose-Any Intensity	One Dose-Light Infections	One Dose-Heavy Infections	Two Doses-Any Intensity	Two Doses-Light Infections	Two Doses-Heavy Infections
							Reported **cure rates** for *Schistosoma mansoni* infection					
Barakat, 2010^s^ [12]	Egypt	588	1 stool, KK	44%	4 weeks	4 weeks	79%	84%	69%	91%	93%	86%
Utzinger, 2000^s^ [21]	Cote d'Ivoire	253	4 stools KK	77%	5 weeks (60 mg/40 mg)	4 weeks	72%	88%	58%	90%	97%	84%
Black, 2009^a^ [13]	Kenya	178	3 stools KK	83%	6 weeks	4–6 weeks	66% (36–82%)			82% (52–92%)		
Picquet, 1998^c^ [18]	Senegal	113	1 stool, KK	87%	6 weeks	4 weeks	43%	53%	33%	76%	71%	81%
Kabatereine, 2003^c^ [14]	Uganda	617	3 stools KK	92%	6 weeks	6 weeks	42%			69%		
							Reported **cure rates** for *Schistosoma haematobium* infection					
Midzi, 2008^s^ [16]	Zimbabwe	675	3 urines, filtration	60%	6 weeks	4 months	89%	90%	86%	97%		
Mduluza, 2001^s^ [15]	Zimbabwe	595	3 urines, filtration	52%	8 weeks	8 weeks	93%			99%		
Tchuem-Tchuente, 2004^s^ [20]	Cameroon	515	2 urines, filtration	42%	3 weeks	6 weeks	83%	87%	69%	82%	85%	70%
N'goran, 2003^s^ [17]	Cote d'Ivoire	354	4 urines, filtration	77%	4 weeks	3 weeks	(74%)*			94%	96%	93%
Sacko, 2009^s^ - [19] Selingue	Mali	256	3 urines, filtration	87%	2 weeks	3 months	57%			58%		
Sacko, 2009^s^ - [19] Koulikoro	Mali	300	3 urines, filtration	95%	2 weeks	3 months	37%			46%		

*estimated the by authors as their effective ‘per treatment’ cure rate.

s Study based on school age participants only;

a Study based on adults only;

c Community-based study.

**Table 3:** Observed reductions in *Schistosoma* infection intensity in selected field studies.

Study [citation]	Country Location	Study Size	Diagnosed with	Local Pre-treatment Prevalence	Two Dose Interval	Follow up interval	One Dose-Any Intensity	One Dose-Light Infections	One Dose-Heavy Infections	Two Doses-Any Intensity	Two Doses-Light Infections	Two Doses-Heavy Infections
							Reported **reduction in ** ***Schistosoma mansoni*** ** infection intensity**					
Barakat, 2010^s^ [12]	Egypt	588	1 stool, KK	44%	4 weeks	4 weeks	71%	34%	87%	74%	34%	89%
Utzinger, 2000^s^ [21]	Cote d'Ivoire	253	4 stools KK	77%	5 weeks (60 mg/40 mg)	4 weeks	80%			89%		
Black, 2009^a^ [13]	Kenya	178	3 stools KK	83%	6 weeks	4–6 weeks	83% (62–91%)					
Picquet, 1998^c^ [18]	Senegal	113	1 stool, KK	87%	6 weeks	4 weeks	71%	30%	88%	88%	56%	96%
Kabatereine 2003^c^ [14]	Uganda	617	3 stools KK	92%	6 weeks	6 weeks	98%			99.6%		
							Reported **reduction in ** ***S. haematobium*** ** infection intensity**					
Midzi, 2008^s^ [16]	Zimbabwe	675	3 urines, filtration	51%	6 weeks	4 months	94%					
Mduluza, 2001^s^ [15]	Zimbabwe	595	3 urines, filtration	52%	8 weeks	8 weeks	96%			92%		
Tchuem-Tchuente, 2004^s^ [20]	Cameroon	515	2 urines, filtration	42%	3 weeks	6 weeks	98%	98%	99%	99%	98%	99.7%
N'goran, 2003^s^ [17]	Cote d'Ivoire	354	4 urines, filtration	77%	4 weeks	3 weeks				97%		
Sacko, 2009^s^ - [19] Selingue	Mali	256	3 urines, filtration	87%	2 weeks	3 months	99%			99%		
Sacko, 2009^s^ - [19] Koulikoro	Mali	300	3 urines, filtration	95%	2 weeks	3 months	98%			99%		

s Study based on school age participants only;

a Study based on adults only;

c Community-based study.

### The relative impact of double treatment on observed cure rates

**Figure 2:**
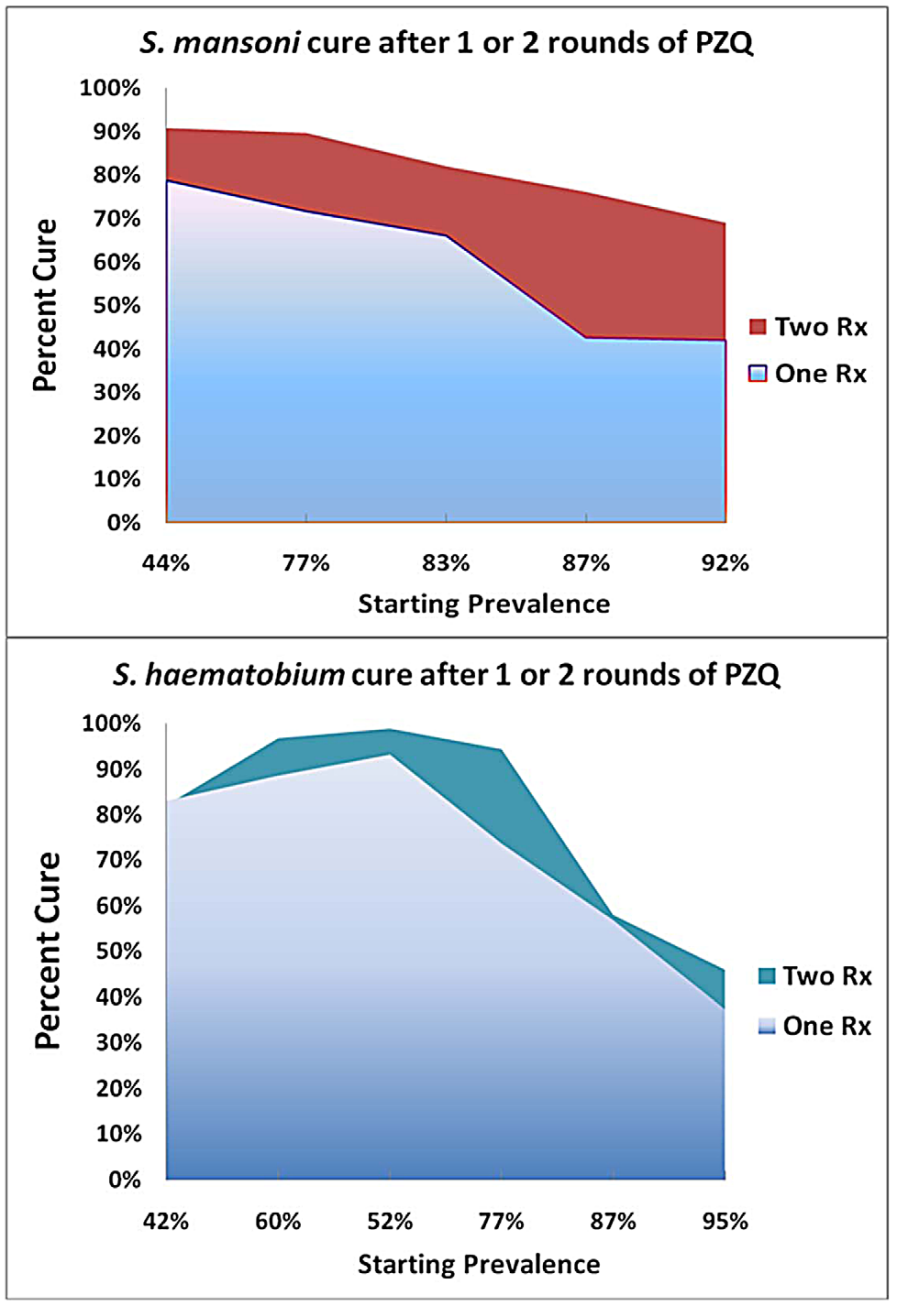
Impact of single vs. repeated praziquantel dosing for cure of *Schistosoma* in high-risk African communities. Upper panel shows efficacy of one- and two-dose regimens for treatment of *S. mansoni* according to the initial pre-treatment infection prevalence of study participants. Lower panel shows the relative efficacy of each treatment schedule for treatment of *S. haematobium*.

For subjects with *S. mansoni* infection, the range of reported cure rates (conversion from positive to negative egg count) was 42% to 79% after a single 40–60 mg/kg dose of praziquantel, and was 69 to 91% after double dosing (Table 2). Cure rates were better in communities having lower initial prevalence (Figure 2), and the odds of cure were consistently higher after the double-dose intervention. With minimal heterogeneity (*I* ^2^ = 0) among the studies, the pooled OR of *S. mansoni* cure after two doses vs. one dose was 3.13, (CI_95%_ 2.59, 3.78). As expected, where intensity-specific data were reported, persons with light intensity infections had higher cure rates than those with heavy infections (Table 2).

Rates of cure for *S. haematobium* infection were more varied, ranging from 37% to 93% after a single-dose treatment and from 46% to 99% after double-dose treatment (Table 2). While cure rates were higher in communities having lower initial community prevalence of infection (Figure 2), double dosing did not show an advantage over single dose treatment of *S. haematobium* in 2/6 locations studied. The heterogeneity score among studies was high (*I* ^2^ = 0.85) indicating considerable variation in results among the *S. haematobium* studies that were included. Where reported, subjects with light intensity infections had higher cure rates than those with heavy infection (Table 2).

### The impact of double treatment on infection intensity

**Figure 3:**
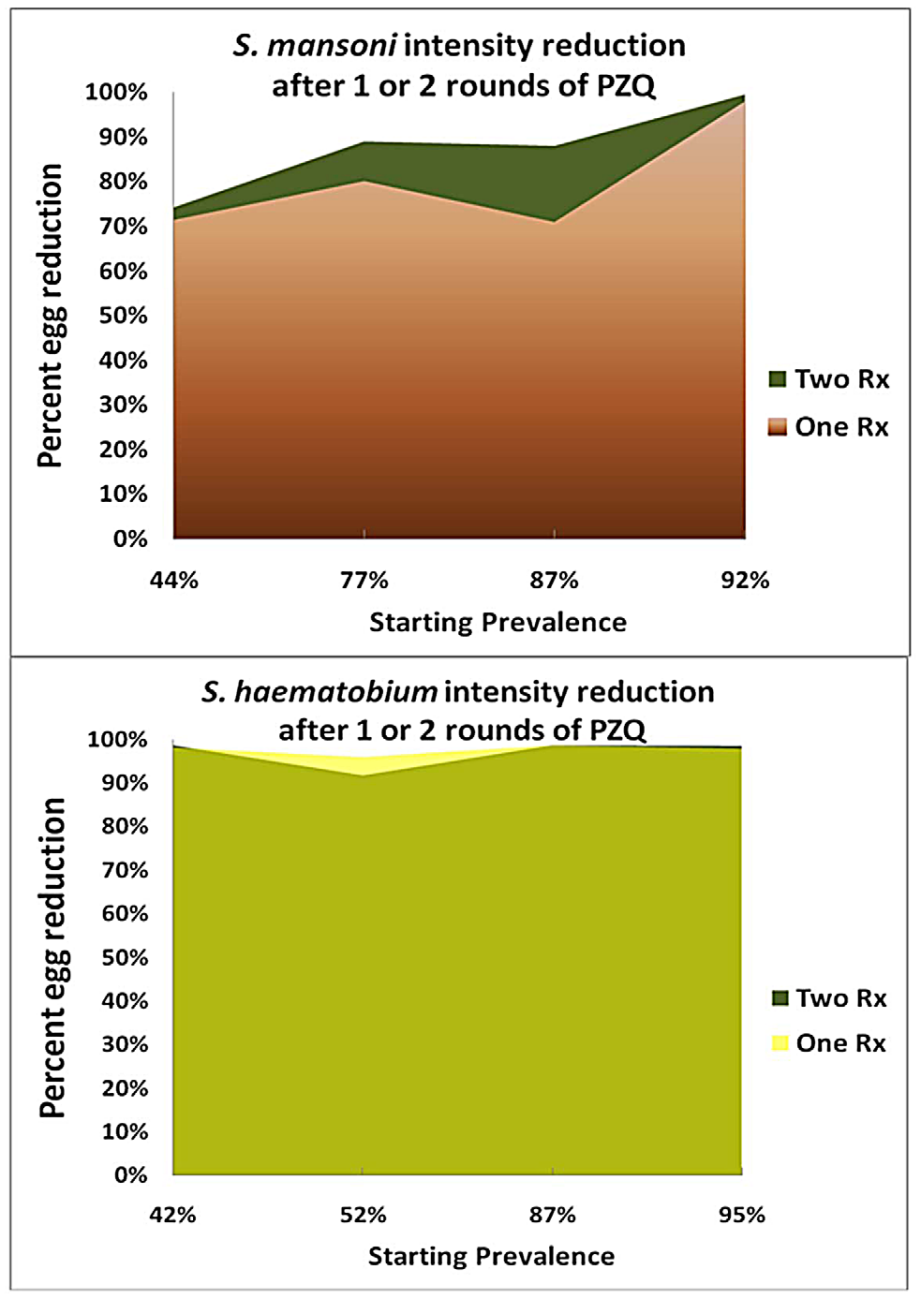
Impact of single vs. repeated praziquantel dosing for intensity (egg output) reduction of *Schistosoma* infection. Upper panel shows reported efficacy of one- and two-dose regimens for treatment of *S.mansoni* according to the initial pre-treatment infection prevalence of study participants. Lower panel shows the relative efficacy for each treatment schedule for treatment of *S. haematobium*.

In examining the effects of single-dose vs. double-dose treatment on infection intensity of *Schistosoma* infection (measured as percent reduction in egg output in stool or urine) the two-dose praziquantel regimen resulted in greater reductions in *S. mansoni* infection than did single dosing. In contrast, there was no apparent advantage to two-dose treatment of *S. haematobium* in this respect (Table 3). Where reported, percent reductions in egg output were proportionately greater (87–96%) for *S. mansoni* subjects with heavy infections than for those with light infections (30–56%), suggesting that the bulk of infections persisting after initial therapy were in the low intensity category. Unlike the influence of local infection prevalence on cure rates (Figure 2), there did not seem to be a consistent effect of pretreatment prevalence on the differences in intensity reduction between one-dose and two-dose regimens (Figure 3). Overall, the 92–99% reduction in *S. haematobium* intensity did not differ between the two treatment groups, while for *S. mansoni*, there were significant further reduction in infection intensity observed in 2/4 of the studies reporting intensity data.

### Modeling the potential long term effects of single-dose and double-dose treatment programs in a high-risk area

**Figure 4:**
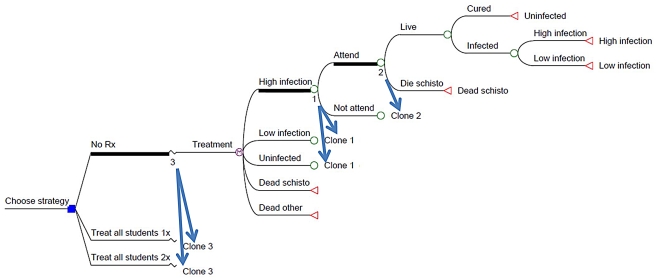
Schematic of the decision tree model used for cost-effectiveness analysis. For each treatment strategy, individuals were cycled annually between three health states (uninfected, light, or heavy infection) or were lost to infection-related death or competing mortality. Transition was dependent on yearly participation with assigned treatment, or, if untreated, on the likelihood of spontaneous increase or reduction of infection without treatment. Input variables for the Markov model are listed in Tables S1 and S2. This is a simplified schematic of the full decision tree. Blue arrows indicate the places in the tree where ‘clones’ of the indicated sub-branches 1, 2, and 3 would be reproduced in the full tree diagram.

**Figure 5:**
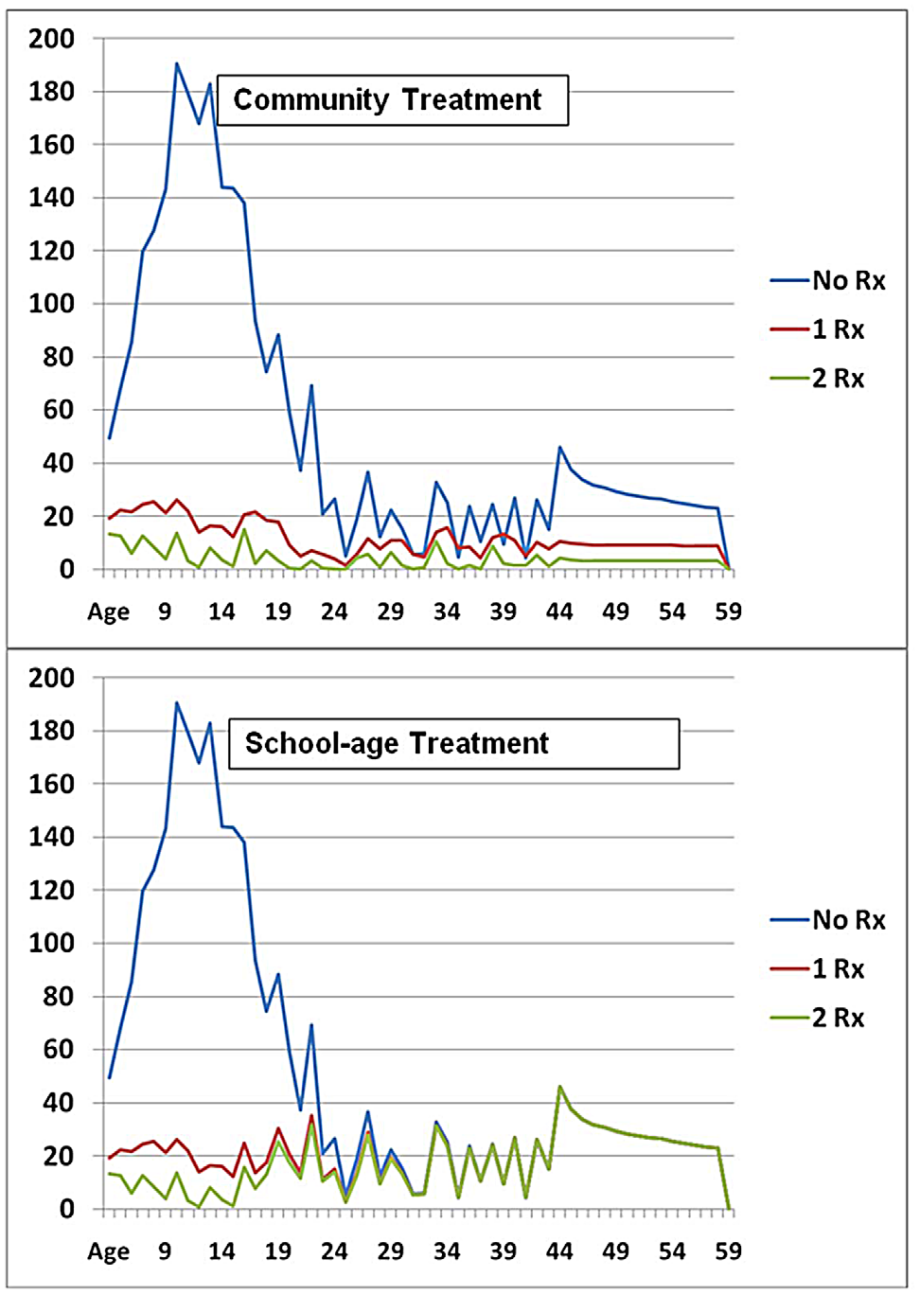
Predicted infection intensity at different ages in a community having continuing *Schistosoma* transmission during control. The upper panel indicates the life path experience with infection intensity (annual mean egg output per specimen) without therapy (No Rx), with single-dose annual therapy (1Rx), with or double-dose annual therapy (2 Rx), and 80% annual adherence in a community-based treatment program when there is continuing transmission of *Schistosoma* during the control intervention. The lower panel indicates the expected lifetime impact of the same regimens in a program treating school-age (5–15 yr) children only.

For this phase of the study, we used a Markov-type computer simulation model to project the likely long-term, lifetime effects of praziquantel treatment implementation in an area with high initial endemicity and risk of continuing local transmission despite continuing treatment [9]. A schematic diagram of the model is shown in Figure 4. Briefly, the model follows a cohort of residents year by year from age 5 to age 60 as they reside in a targeted treatment community and experience repeated treatments either through school age (5 to 15 yr), or throughout the entire period, in a community-wide program. The model included the possibility of individual non-adherence to therapy in any given year. Treatment adherence levels, drug efficacy, age-related risk of infection/reinfection, and costs input to the model were drawn from published experience in similar mass drug administration programs for schistosomiasis and other NTDs (Table S1). In the simulation, comparisons were made between the results for no therapy (No Rx), *vs.* two different treatment strategies in which residents were either offered a single-dose treatment each year, or a double-dose regimen each year. Figure 5 shows the model's projected *S. haematobium* infectious burden (egg output) at different ages for participating communities that received No Rx or single- or double-dose treatment, at an average 80% level of annual adherence to treatment.

### The impact of coverage on infection and lifetime burden

Of note is the substantial reduction of infection levels in the 5 to 25 year age groups whether the program was limited to school age coverage (lower panel) or it used community-directed treatment of children and adults (upper panel). However, in the Markov model simulations, if coverage were limited to school age residents, then infection/reinfection in adult years remained a persistent problem (Figure 5, lower panel). Tables 4 and 5 summarize the projected relative impact of the two treatment strategies in infection duration and cumulative lifetime burden as compared to the No Rx situation. Without treatment, local residents were estimated to spend nearly 20 years with infection and 7 years with heavy infection, experiencing 2973 egg-years of infection intensity. These numbers were substantially reduced with therapy– to 12 to 13 years of infection in a school age treatment program, and only 6 to 9 years of infection during the course of a community-based program. Corresponding reductions in lifetime infectious burden are 61–67% for school age treatment and 78–92% with community therapy.

**Table 4:** Incremental cost-effectiveness of a two dose regimen compared to a single dose regimen in a continuing *community-wide* mass drug campaign.

Strategy	Life years spent infected by species^a^	Years spent with heavy infection	Cumulative lifetime cost^b^	Incremental Cost	Lifetime Egg-Years^b^	Lifetime QALYs^b^	Incremental cost-effectiveness^c^		
							$ per infection year averted	$ per egg year averted	$ per QALY gained
Without treatment	19.7	6.8	$0.00	–	2973	26.96	–	–	–
One dose per annual treatment, ages 5–55	9.1 Sm 7.4 Sh	1.1 Sm 0.9 Sh	$23.01	$23.01	657 Sm 529 Sh	27.46 Sm 27.47 Sh	$2.17 Sm $1.87 Sh	$0.01 Sm $0.009 Sh	$47.90 Sm $45.58 Sh
Two doses per annual treatment, ages 5–55	5.6 Sm 5.7 Sh	0.2 Sm 0.2 Sh	$46.03	$23.02	223 Sm 229 Sh	27.52 Sm 27.52 Sh	$6.58 Sm $13.54 Sh	$0.053 Sm $0.077 Sh	$291.07 Sm $432.76 Sh

a Abbreviations: Sm, *Schistosoma mansoni* infection; Sh, *Schistosoma haematobium* infection; QALY, Quality-adjusted Life Year.

b Time discounted at 3% per annum.

c Relative to strategy in the row immediately above.

**Table 5:** Incremental cost-effectiveness of a two dose regimen compared to a single dose regimen in a continuing *school-age* mass drug campaign.

Strategy	Life years spent infected by species^a^	Years spent with heavy infection	Cumulative lifetime cost^b^	Incremental Cost	Lifetime Egg-Years^b^	Lifetime QALYs^b^	Incremental cost-effectiveness^c^		
							$ per infection year averted	$ per egg year averted	$ per QALY gained
Without treatment	19.7	6.8	$0.00	–	2973	26.96	–	–	–
One dose per annual treatment, ages 5–16	13.1 Sm 12.5 Sh	2.1 Sm 2.1 Sh	$7.55	$7.55	1160 Sm 1107 Sh	27.39 Sm 27.40 Sh	$1.14 Sm $1.05 Sh	$0.0042 Sm $0.0040 Sh	$17.76 Sm $17.18 Sh
Two doses per annual treatment, ages 5–16	11.7 Sm 11.7 Sh	1.7 Sm 1.7 Sh	$15.11	$7.55	969 Sm 966 Sh	27.44 Sm 27.44 Sh	$5.39 Sm $9.44 Sh	$0.039 Sm $0.053 Sh	$152.95 Sm $210.83 Sh

a Abbreviations: Sm, *Schistosoma mansoni* infection; Sh, *Schistosoma haematobium* infection; QALY, Quality-adjusted Life Year.

b Time discounted at 3% per annum.

c Relative to strategy in the row immediately above.

### The impact of double-dose treatment

In the simulation, for each coverage scenario, repeated years of double treatment were projected to provide incremental improvements over single treatment in terms of reducing the number of years spent infected, the cumulative egg-years, and in improving cumulative quality-of-life (measured as infection-related QALYs). Most remarkably, double-dose treatment on a community-wide basis was projected to nearly eliminate the time spent with heavy infection (0.2 yr instead of 6.8 yr per lifetime, Table 4).

Different projections reported for *S. mansoni* and *S. haematobium* in terms of these outcomes (Tables 4 and 5) were based on the differences observed in average drug efficacy found in our systematic review (Tables 2 and 3). Community-wide therapy (given over more years of life) resulted in greater cumulative differences between double-dose therapy and single-dose therapy. Nevertheless, because of continuing re-exposure, long-term double-dosing did not entirely eliminate the risk of infection, with a remaining 5–6 years spent infected and 223–229 egg-years accumulation with double-dose programs (Table 4). In each coverage scenario, double-dosing was twice as expensive as single dosing, whereas community-wide treatment was approximately three times as expensive as school age therapy at either dosing frequency. Because of the relatively greater incremental benefit of repeated dosing for *S. mansoni* (Tables 2 and 3), our models estimated greater double-dose reductions in lifetime egg burden and in time spent infected for persons infected with *S. mansoni* when compared to those with *S. haematobium* (Tables 4 and 5).

### Improvements in quality of life and incremental cost-effectiveness estimates

Estimates for improvement in quality of life (time-discounted QALYs) were most closely linked to reductions in time spent with heavy infection (Tables 4 and 5). The QALY gains were greatest between the No Rx setting and either treatment strategy. Estimated cost per discounted QALY gained by single-dosing were $17–18 for school age coverage programs, and $46–48 for community-wide programs. The *incremental* QALY benefits of double-dosing came at a higher price– $153–211 for each additional QALY gained by expanding to double-dosing in school age programs, and $291–433 per additional QALY gained in community-wide programs (Tables 4 and 5).

In estimating total expected life years for individuals within each strategy, there was only a small difference between no treatment (54.870 years), treatment with 1 dose of PZQ (54.982 years), and treatment with 2 doses of PZQ (54.998 years). This was due to the fact that the mortality rate from schistosomiasis is relatively small [3], and regardless of intervention strategy the global impact of treatment on mortality is consequently also small. Furthermore, because there are many competing risks for early mortality (assumed to be even in each strategy), cumulative benefits in terms of lifetime survival are reduced.

### Sensitivity analysis of model predictions

**Figure 6:**
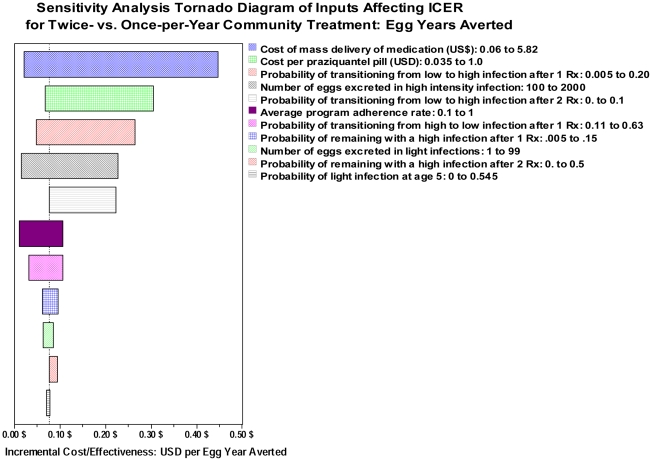
Sensitivity analysis of ICER estimates for egg output reduction in community-based therapy of *S. haematobium*. Shown are the 11 most influential inputs to the Markov decision tree model and the effects of their variation on $US cost per cumulative egg-year averted when calculating the incremental cost effectiveness of single-dose *vs.* double-dose treatment regimens in community-based programs. The base case analysis from Table 4 is indicated by the vertical dotted line.

**Figure 7:**
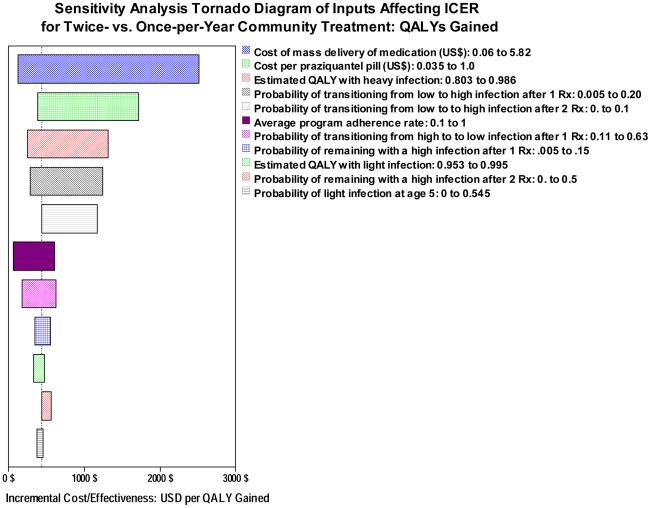
Sensitivity analysis of ICER estimates for QALYs gained in community-based therapy of *S. haematobium*. Shown are the 11 most influential inputs to the model and the effects of their variation on $US cost per lifetime QALY gained when calculating the incremental cost effectiveness of single-dose *vs.* double-dose treatment regimens in community-based programs. The base case analysis from Table 4 is indicated by the vertical dotted line.

To provide a more general view of our model's predictions, we performed sensitivity analysis to determine how variation in each individual model input would influence the projected ICER measured between double-dose and single-dose strategies. Figure 6 shows a tornado diagram indicating the model parameters that were most influential in determining the double-dose/single-dose ICER for cost per egg year averted. Here, the six most influential model inputs were found to be the cost of drug delivery, the cost of drug, the yearly chance of moving from light to heavy infection (a measure of local transmission intensity) after either single-dose or double-dose treatment, the number of eggs excreted with heavy intensity infection, and the level of program adherence. Figure 7 shows a similar display of input factors most strongly influencing the ICER in terms of costs per QALY gained. Here, the six most influential factors were quite comparable: The cost of drug delivery, the cost of drug, the estimated QALY value associated with the heavy infection state, the probability of moving from light to heavy infection after either type of therapy, and the program adherence.

## Discussion

Results of our systematic review of treatment outcomes suggest there were significant improvements to be gained in terms of *S. mansoni* infection outcomes by implementing a double-dose regimen in which patients were retreated 2–8 weeks after their initial PZQ dose. By contrast, on a one-time basis, a single-dose PZQ treatment was very nearly as effective as the more aggressive repeated dose treatment of *S. haematobium*. Published information on the long-term multi-year impact of such repeat-dose strategies was not available. However, we were able to use model-based simulations to estimate the long-term programmatic costs and efficacy of single and double-dose strategies in a high *Schistosoma*-infection transmission setting. As expected, the more limited programs were projected to be proportionately less expensive, but these were less effective as well. The cost-effectiveness analysis did not identify a dominant or ‘optimal’ strategy. Rather, although the difference in relative cost-effectiveness between one and two-dose strategies was sensitive to drug and delivery costs and community participation, increased investment was reliably associated with increased benefits in our multi-year scenarios.

The analysis demonstrated that treating children or communities with two sequential doses of PZQ could be a cost-effective treatment plan for areas with high initial prevalence of *S. mansoni* or *S. haematobium*, even in the face of ongoing transmission risk. Another perceived advantage to a second-dose treatment plan (not explored in our simulation) is that treatment may reach more community members by giving people a second opportunity to be treated at least once if they had missed their first dose. In this fashion, more untreated community members will eventually be reached, having a larger impact on community prevalence over time. Debate continues about whether there is a recognized infection intensity or infection duration threshold below which the risk for disease from *Schistosoma* infection becomes negligible [1], [3], [36]. If moderate to heavy infection intensity is the driver of disease causation in schistosomiasis, then double-dosing will more effectively reduce disease prevalence by reducing the lifetime experience with heavy infection (Tables 4 and 5). If risk of morbidity is not strictly intensity dependent (e.g., because of host immunopathological responses to all levels of infection [37]–[41]), then only prevention of infection will fully eliminate disease. However, there may be an effect of double dosing that was not studied in our simulations–If double-dosing could reliably reduce local transmission, then it could exert a non-linear, or ‘tipping point’ effect in reducing the lifetime risk of reinfection, making the double-dose strategy the much more effective strategy to eliminate all forms of infection-related disease. Under such conditions, the second-dose treatment plan could prove not only cost-efficient, but also cost-saving, and so become the dominant strategy for treatment in such areas.

Our structured review indicates that repeated praziquantel appears to provide greater relative benefits in terms of increased cure rates and increased reduction in infectious burden for persons having *S. mansoni* infection as compared to those having *S. haematobium* infection. The reasons for this difference are presently unknown. The difference in response may reflect inherent differences in susceptibility of the two parasite species to praziquantel, with *S. mansoni* being slightly less responsive [4]. Alternatively, environmental factors that favor transmission of *S. mansoni* (*e.g.*, permanent water bodies *vs.* smaller transient ponds [42], [43]) may allow more continuous transmission, and a greater likelihood that patients will have immature larvae that will not be effectively eliminated by a single-dose annual treatment [11].

There are limitations to our analysis. Our modeling was facilitated by certain simplifying assumptions listed in Table 1. Admittedly, this makes for an artificial picture of program implementation compared to the real world experience in schistosomiasis-endemic areas. Nevertheless, we think that the analysis provides a realistic ‘worst-case’ scenario, which is still very useful in comparing the potential incremental benefits of the double-dose treatment strategy. It is unlikely that a control program would continue with a single strategy for a 55 year period of time. However, our simulation of program performance in difficult-to-treat, high prevalence, high-transmission areas [6], [9], [44] indicates the maximal relative impact that a double-dose program might have in the face of ongoing transmission. Although it is likely that other behavioral and sanitation changes will occur as programs mature, until their beneficial impact (in combination with PZQ mass therapy) is better defined, we can use the current model's estimates as a floor value for the cost-effectiveness of single- and double-dose strategies. In anticipation of the difficulties in repeatedly targeting those who have exposure to reinfection and in having community members adhere to a multi-year treatment plan, the long-term programs should probably be a collaborative effort, with community-health care workers functioning within a community directed treatment program [45]–[47].

Our data for age-specific risk of infection, reinfection, and spontaneous loss of infection were taken from an area of Kenya that is highly endemic for *S. haematobium*, and it is likely that post-treatment outcomes and thus cost-effectiveness would proportionately better in areas with lower risk of reinfection. We have included as supplemental material the full tables of our infection inputs to the decision-tree model, so that where other age-specific data are known, these could be substituted to provide a more location-specific cost-benefit analysis.

The optimal timing interval of a second PZQ dose also remains uncertain. The studies included in our review provided retreatment at a range of 2–8 weeks after the initial PZQ dose (Table 2). Additional testing is therefore recommended to define the optimal second dosing interval. Another limitation is the absence of data on the effect that repeated treatment has on the probability of dying from schistosomiasis. The mortality risks used in this paper were predominantly from untreated populations [3]. Theoretically, repeated treatments over time could impact survival, but the size of this effect is currently unknown.

Finally, the decision point value for the ‘willingness-to-pay’ for such a program may vary. Suggested thresholds have ranged from 1 to 3 times the per-capita GDP per additional quality-adjusted life-year [48]–[51]. Kenya's GDP per capita was $US 1,600 in 2010 [52]. Therefore, both single- and double- treatment methods were judged to be very cost effective as the respective ICERs were far less than $US 1,600 (1 times per-capita GDP) per QALY gained, at $17 per incremental QALY for single-dose *vs.* no therapy, and then $210 per incremental QALY for double-dose *vs.* single-dose therapy in school age treatment programs (Table 5). For community-wide programs the respective amounts were $45 and $433 for additional QALYs gained by single dose therapy and then the more aggressive double-dose therapy. However, these sums of money, spread over a lifetime, may not seem feasible to present day national policymakers who have to allocate scarce resources to a host of competing needs. For contrast, the ICER for sulfamethoxazole-trimethoprim antibiotic prophylaxis of opportunistic infections (versus no treatment) among HIV+ patients in Cote D'Ivoire has been estimated at $240 per life year gained [49]. For blood pressure control in low-income Southeast Asia, the *annual* cost for each disability-adjusted life year (DALY) gained by basic antihypertensive treatment are estimated at $36/year [50], which translates to $540–$900 time-discounted total cost/DALY (in that setting) for a regimen of 15–25 years of preventive drug therapy given over a person's lifetime.

In direct terms, a country like Kenya, with ∼480,000 children entering school-age in schistosomiasis-risk areas each year, would need to commit approximately $4 million in funds each year to maintain an annual single-dose program for school age therapy, and $18.4 million for single-dose community coverage. Prices would be double for twice-a-year treatment programs, although their attendant benefits should be seen to be worth the extra investment, particularly if transmission interruption is achieved in some areas, thereby reducing the required duration of the program in those locales. Further, if economies of scale (and economies of scope with integrated NTD management) can reduce the drug and delivery costs, these estimates would be substantially reduced.

Ultimately, collaboration among local agencies will be needed to determine the strength of current resources. These, along with an informed view of the expected long-term benefits of schistosomiasis control and good knowledge of the size of the target population, will ensure overall program sustainability and thus long-term effectiveness for these types of population-based schistosomiasis control programs.

## Supporting Information

Table S1 **Parameter values for the cost-effectiveness Markov model, indicating base case values and the ranges used for sensitivity analyses.**
(DOC)

Table S2 **The natural history of untreated schistosomiasis, given as age-specific annual probabilities of transition between different levels of infection intensity in the absence of any treatment.**
(DOC)

Model S1 **Program file for the decision-tree model of costs and benefits of a** ***Schistosoma haematobium*** **treatment program if implemented on a community-wide basis.** The file format is a ‘package’ file, containing necessary input tables and variables, to be used with TreeAge software, (Tree-Age Pro 2009, version 1.0.2, TreeAge Software, Inc. Williamstown MA).
(ZIP)
