# Supplementary material for: Utility of Repeated Praziquantel Dosing in the Treatment of Schistosomiasis in High-Risk Communities in Africa: A Systematic Review
Source: PLoS Negl Trop Dis. 2011 Sep 20;5(9):e1321. doi: 10.1371/journal.pntd.0001321 (PMC3176745; doi:10.1371/journal.pntd.0001321)
Supplement: Table S1 — Parameter values for the cost-effectiveness Markov model, indicating base case values and the ranges used for sensitivity analyses. (DOC) [file pntd.0001321.s001.doc]

Table S1. Parameter values for the cost-effectiveness Markov model, indicating base case values and the ranges used for sensitivity analyses.

|  |
| --- |

| **Parameter**  **(coded software input name)** | **Baseline Value** | **Range for Sensitivity Analysis** | **Reference(s)** |
| --- | --- | --- | --- |
| Probability of moderate or heavy infection at age 5 (phigh_age5) | 0.033 | 0 – 0.219 | King et al., 1988  Muchiri et al., 1996  King et al., 2000 |
| Probability of light infection at age 5 (plow_age5) | 0.121 | 0 – 0.545 | King et al., 1988  Muchiri et al., 1996  King et al., 2000 |
| Program attendance rate (p_attend) | 0.8 | 0.10 – 1 | King et al., 1991  Chan et al., 1998  Chan et al., 1999  King et al., 2000 |
| Probability of dying from high intensity *Schistosoma* infection (pdead_schistoHi) | 0.0005 | 0 – 0.003 | Van der Werf & de Vlas, 2001 |
| Probability of dying from active or recent low intensity *Schistosoma* infection (pdead_schistolono) | 0.00000026 | 0 – 0.000001 | Van der Werf & de Vlas, 2001 |
| For those with moderate-heavy infection, age-specific annual probability of remaining in the same state without therapy (pH2H_0Rx) | Table thi_hi | - | King et al., 1988  Muchiri et al., 1996  King et al., 2000 |
| For those with moderate-heavy infection, probability of remaining in the same state after a single-dose round of therapy (pH2H_1Rx) | 0.0785 | 0.005 – 0.15 | King et al., 1988  Muchiri et al., 1996  King et al., 2000  Midzi et al., 2008  Utzinger et al., 2000  Barakat & Morshedy, 2010 |
| For those with moderate-heavy infection, probability of remaining in the same state after a double-dose round of therapy (pH2H_2Rx) | 0 | 0 – 0.5 | N'Goran, et al., 2003  Barakat & Morshedy, 2010 |
| For those with moderate-heavy infection, age-specific annual probability of transitioning to low intensity infection without therapy (pH2L_0Rx) | Table thi_low |  | King et al., 1988  Muchiri et al., 1996  King et al., 2000 |
| For those with moderate-heavy infection, probability of transitioning to low intensity infection after a single-dose round of therapy (pH2L_1Rx) | 0.42 Sm  0.26 Sh | 0.22 – 0.66  0.11-0.63 | Base case = Median values from Table 2  Range for sensitivity = Range in Table 2 |
| For those with moderate-heavy infection, probability of transitioning to low intensity infection after a double-dose round of therapy (pH2L_2Rx) | 0.16 Sm  0.11 Sh | 0.02 – 0.31  0.03 – 0.54 | Base case = Median values from Table 2  Range for sensitivity = Range in Table 2 |
| For those with low intensity infection, age-specific annual probability of transitioning to moderate-heavy infection without therapy (pL2H_0Rx) | Table tlow_hi |  | King et al., 1988  Muchiri et al., 1996  King et al., 2000 |
| For those with low intensity infection, probability of transitioning to moderate-heavy infection after a single-dose round of therapy (pL2H_1Rx) | 0.103 | 0.005 – 0.20 | King et al., 1988  Muchiri et al., 1996  King et al., 2000  Midzi et al., 2008  Barakat & Morshedy, 2010 |
| For those with low intensity infection, probability of transitioning to moderate-heavy infection after a double-dose round of therapy (pL2H_2Rx) | 0 | 0 – 0.10 | N'Goran, et al., 2003  Barakat & Morshedy, 2010 |
| For those with low intensity infection, age-specific annual probability of remaining in the same state without therapy (pL2L_0Rx) | Table tlow_low |  | King et al., 1988  Muchiri et al., 1996  King et al., 2000 |
| For those with low intensity infection, probability of remaining in the same state after a single-dose round of therapy (pL2L_1Rx) | 0.16 Sm  0.12 Sh | 0.12–0.47 Sm  0.07-0.13 Sh | Base case = Median values from Table 2  Range for sensitivity = Range in Table 2 |
| For those with low intensity infection, probability of remaining in the same state after a double-dose round of therapy (pL2L_2Rx) | 0.07 Sm  0.10 Sh | 0.03 – 0.29 Sm  0.04-0.15 Sh | Base case = Median values from Table 2  Range for sensitivity = Range in Table 2 |
| For those uninfected, age-specific annual probability of developing moderate-heavy infection (puninf_hi) | 0 | -- | King et al., 1988  Muchiri et al., 1996  King et al., 2000 |
| For those uninfected, age-specific annual probability of developing low intensity infection (puninf-low) | Table tuninf_low |  | King et al., 1988  Muchiri et al., 1996  King et al., 2000 |
| Cost of delivery of individual treatment (Cost_delivery) | 0.811 | 0.06 – 5.82 | Goldman et al., 2007 Based on delivery costs for ongoing lymphatic filariasis mass-drug administration programs. The range for sensitivity encompasses the range of basic financial and more comprehensive economic costs |
| Cost per PZQ pill (Cost_pill) | 0.07 | 0.035 – 1.00 | Fenwick et al., 2009 Lowest value set at $0.035, presuming that cost of drug may get cheaper (by half), and highest at $1 per pill, presuming brand-name cost of drug. |
| Mean number of eggs excreted by moderate to heavy infections (No_eggs_hi) | 379 | 100 – 2000 | King et al., 1988  Nsowah-Nuamah et al., 2004 |
| Mean number of eggs excreted in light infections (no_eggs_lo) | 33 | 1 – 99 | King et al., 1988  Nsowah-Nuamah et al., 2004 |
| Estimated QALY with moderate-to heavy infection (Uhi) | 0.90 | 0.803 – 0.986 | King et al., 2005  Jia et al., 2007  King & Dangerfield-Cha, 2008  Finkelstein et al., 2008  King, 2010 |
| Estimated QALY for light infection (Ulow) | 0.986 | 0.953 – 0.995 | King et al., 2005  Jia et al., 2007  King & Dangerfield-Cha, 2008  Finkelstein et al., 2008  King, 2010 |
