# Supplementary material for: Utility of Repeated Praziquantel Dosing in the Treatment of Schistosomiasis in High-Risk Communities in Africa: A Systematic Review
Source: PLoS Negl Trop Dis. 2011 Sep 20;5(9):e1321. doi: 10.1371/journal.pntd.0001321 (PMC3176745; doi:10.1371/journal.pntd.0001321)
Supplement: Table S2 — The natural history of untreated schistosomiasis, given as age-specific annual probabilities of transition between different levels of infection intensity in the absence of any treatment. (DOC) [file pntd.0001321.s002.doc]

**Table S2: Age-specific annual probabilities of transition between different levels of infection intensity without therapy^a^**

| Age | thi_hi | thi_low | tlow_hi | tlow_low | tuninf_low |
| --- | --- | --- | --- | --- | --- |
| 5 | 0.984 | 0.000 | 0.694 | 0.290 | 0.143 |
| 6 | 0.984 | 0.000 | 0.253 | 0.731 | 0.259 |
| 7 | 0.984 | 0.000 | 0.176 | 0.794 | 0.000 |
| 8 | 0.984 | 0.000 | 0.355 | 0.629 | 0.325 |
| 9 | 0.984 | 0.000 | 0.068 | 0.916 | 0.167 |
| 10 | 0.988 | 0.000 | 0.139 | 0.825 | 0.000 |
| 11 | 0.988 | 0.000 | 0.428 | 0.560 | 0.367 |
| 12 | 0.934 | 0.053 | 0.000 | 0.988 | 0.000 |
| 13 | 0.933 | 0.054 | 0.000 | 0.926 | 0.000 |
| 14 | 0.988 | 0.000 | 0.138 | 0.850 | 0.234 |
| 15 | 0.752 | 0.228 | 0.000 | 0.980 | 0.066 |
| 16 | 0.980 | 0.000 | 0.031 | 0.825 | 0.000 |
| 17 | 0.922 | 0.058 | 0.000 | 0.980 | 0.455 |
| 18 | 0.680 | 0.300 | 0.000 | 0.483 | 0.000 |
| 19 | 0.707 | 0.273 | 0.000 | 0.980 | 0.215 |
| 20 | 0.973 | 0.000 | 0.116 | 0.644 | 0.000 |
| 21 | 0.626 | 0.346 | 0.000 | 0.766 | 0.000 |
| 22 | 0.567 | 0.406 | 0.000 | 0.752 | 0.000 |
| 23 | 0.973 | 0.000 | 0.313 | 0.660 | 0.093 |
| 24 | 0.193 | 0.780 | 0.000 | 0.612 | 0.000 |
| 25 | 0.972 | 0.000 | 0.079 | 0.743 | 0.000 |
| 26 | 0.022 | 0.950 | 0.000 | 0.407 | 0.000 |
| 27 | 0.972 | 0.000 | 0.229 | 0.743 | 0.132 |
| 28 | 0.972 | 0.000 | 0.215 | 0.757 | 0.097 |
| 29 | 0.247 | 0.724 | 0.000 | 0.413 | 0.000 |
| 30 | 0.971 | 0.000 | 0.101 | 0.870 | 0.189 |
| 31 | 0.579 | 0.391 | 0.000 | 0.814 | 0.000 |
| 32 | 0.021 | 0.950 | 0.000 | 0.607 | 0.000 |
| 33 | 0.971 | 0.000 | 0.000 | 0.971 | 0.022 |
| 34 | 0.971 | 0.000 | 0.295 | 0.676 | 0.310 |
| 35 | 0.713 | 0.254 | 0.000 | 0.799 | 0.000 |
| 36 | 0.017 | 0.950 | 0.000 | 0.301 | 0.000 |
| 37 | 0.967 | 0.000 | 0.401 | 0.566 | 0.040 |
| 38 | 0.474 | 0.493 | 0.000 | 0.067 | 0.000 |
| 39 | 0.967 | 0.000 | 0.500 | 0.467 | 0.267 |
| 40 | 0.011 | 0.950 | 0.000 | 0.912 | 0.000 |
| 41 | 0.961 | 0.000 | 0.243 | 0.082 | 0.000 |
| 42 | 0.011 | 0.950 | 0.000 | 0.961 | 0.040 |
| 43 | 0.961 | 0.000 | 0.429 | 0.532 | 0.121 |
| 44 | 0.468 | 0.492 | 0.000 | 0.795 | 0.000 |
| 45 | 0.951 | 0.000 | 0.519 | 0.432 | 0.091 |
| 46 | 0.591 | 0.360 | 0.140 | 0.591 | 0.063 |
| 47 | 0.591 | 0.360 | 0.140 | 0.591 | 0.063 |
| 48 | 0.591 | 0.360 | 0.140 | 0.591 | 0.063 |
| 50 | 0.574 | 0.360 | 0.140 | 0.574 | 0.063 |
| 55 | 0.547 | 0.360 | 0.140 | 0.547 | 0.063 |
| 60 | 0.547 | 0.360 | 0.140 | 0.504 | 0.063 |

^a^Abbreviations: thi_hi = persistence of moderate to heavy infection; thi_low = transition from moderate-heavy infection to low intensity infection; tlow_hi = transition from low intensity infection to moderate or heavy infection; tlow_low = persistence of low intensity infection; tuninf_low = transition from uninfected state to low intensity infection. Not shown, model transition probabilities hi_uninf and uninf_hi were zero. Transition probability low_uninf was set to 1-[low_hi + low_low]. Data are derived from age-specific individual treatment program results reported in references [53], [54] and [55].
